# Supplementary material for: Complex‐centric proteome profiling by SEC‐SWATH‐MS
Source: Mol Syst Biol. 2019 Jan 14;15(1):e8438. doi: 10.15252/msb.20188438 (PMC6346213; doi:10.15252/msb.20188438)
Supplement: Supplementary file 6 — Dataset EV5 [file MSB-15-e8438-s006.zip › feature_plots_corum/300.pdf]

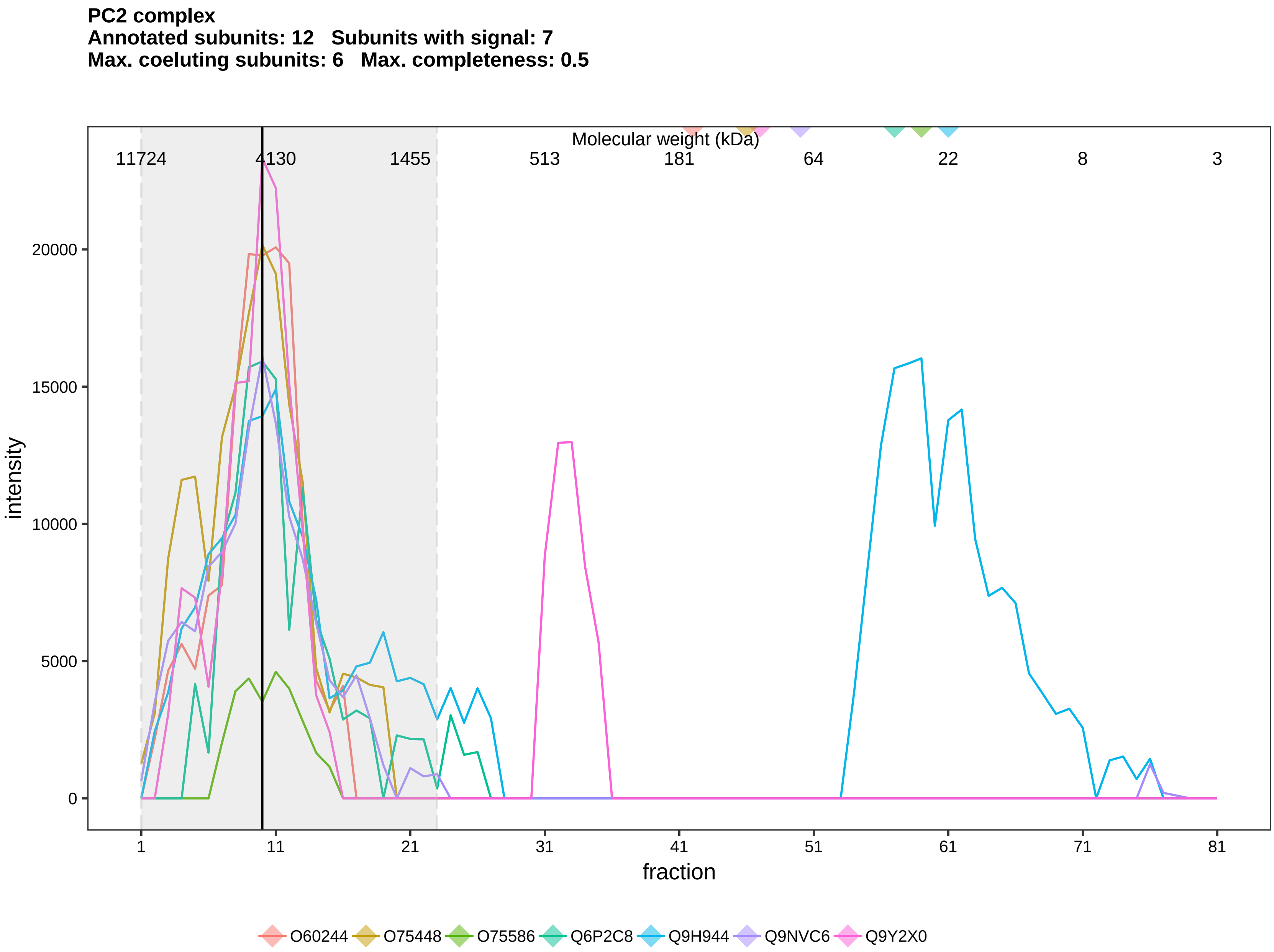

PC2 complex
Annotated subunits: 12   Subunits with signal: 7
Max. coeluting subunits: 6   Max. completeness: 0.5
Molecular weight (kDa)
11724
4130
1455
513
181
64
22
8
3
intensity
20000
15000
10000
5000
0
1
11
21
31
41
51
61
71
81
fraction
O60244
O75448
O75586
Q6P2C8
Q9H944
Q9NVC6
Q9Y2X0
